# How to Defeat Multidrug-Resistant Bacteria in Intensive Care Units. A Lesson from the COVID-19 Pandemic. Prevention, Reservoirs, and Implications for Clinical Practice

**DOI:** 10.7150/ijms.88519

**Published:** 2024-01-12

**Authors:** Anna Woźniak, Jarosław Janc, Lidia Łysenko, Patrycja Leśnik, Natalia Słabisz, Monika Oleksy-Wawrzyniak, Izabella Uchmanowicz

**Affiliations:** 1 Department of Nursing and Midwifery, Wroclaw Medical University, Wroclaw, Poland.; 2 Department of Anaesthesiology and Intensive Therapy, Hospital of Ministry of the Interior and Administration, Wroclaw, Poland.; 3 Department of Anaesthesiology and Intensive Therapy, Wroclaw Medical University, Wroclaw, Poland.; 4 Department of Microbiology, Wroclaw Medical University, Wroclaw, Poland.; 5 Department of Laboratory Diagnostics, 4th Military Clinical Hospital, Wroclaw, Poland.; 6 Department of Pharmaceutical Microbiology and Parasitology, Wroclaw Medical University, Wroclaw, Poland.

**Keywords:** COVID-19, drug resistance, bacterial, molecular epidemiology, mortality, primary prevention

## Abstract

**Background:** Intensive care unit (ICU) patients are at high risk of infection due to multiple invasive procedures, malnutrition, or immunosuppression. The rapid increase in infections with multidrug-resistant organisms (MDRO) during the COVID-19 pandemic caused a dilemma, as the rules of the sanitary regime in ICU rooms were strictly adhered to in the prevailing epidemiological situation. The combat to reduce the number of infections and pathogen transmission became a priority for ICU staff. This study aimed to assess whether eliminating environmental reservoirs and implementing improved procedures for patient care and decontamination and washing equipment in the ICU reduced the incidence of infections caused by MDR strains.

**Material and methods:** The study retrospectively analyzed data in the ICU during the COVID-19 pandemic. The samples were collected based on microbiological culture and medical records in the newly opened ICU (10 stations) and hospital wards where COVID-19 patients were hospitalized. Environmental inoculations were performed during the COVID-19 pandemic every 4-6 weeks unless an increase in the incidence of infections caused by MDR strains was observed. Through microbiological analysis, environmental reservoirs of MDR pathogens were identified. The observation time was divided into two periods, before and after the revised procedures. The relationship between isolated strains of Klebsiella pneumoniae NDM from patients and potential reservoirs within the ICU using ERIC-PCR and dice methods was analyzed.

**Results:** An increased frequency of infections and colonization caused by MDRO was observed compared to the preceding years. A total of 23,167 microbiological tests and 6,985 screening tests for CPE and MRSA bacilli were collected. The pathogen spread was analyzed, and the findings indicated procedural errors. Assuming that the transmission of infections through the staff hands was significantly limited by the restrictive use of personal protective equipment, the search for a reservoir of microorganisms in the environment began. MDR strains were grown from the inoculations collected from the hand-wash basins in the wards and from inside the air conditioner on the ceiling outside the patient rooms. New types of decontamination mats were used in high-risk areas with a disinfectant based on Glucoprotamine. Active chlorine-containing substances were widely used to clean and disinfect surfaces.

**Conclusions:** Infections with MDR strains pose a challenge for health care. Identification of bacterial reservoirs and comprehensive nursing care significantly reduce the number of nosocomial infections.

## Introduction

In March 2022, the European Centre for Disease Prevention and Control issued a report on European antimicrobial resistance surveillance 1,2. The presented information is based on antimicrobial resistance (AMR) data from invasive isolates reported to the Central Asian and European Surveillance of Antimicrobial Resistance (CAESAR) network and the European Antimicrobial Resistance Surveillance Network (EARS-Net) in 2021 (data referring to 2020). Twelve countries and Kosovo reported data to CAESAR, while 29 countries, including all from the European Union (EU) and two from the European Economic Area (EEA) (Iceland and Norway), reported data to EARS-Net.

Nearly half of all healthcare-associated infections in hospitals are related to the intensive care unit (ICU) stay, where approximately 20% of critically ill patients become infected 3. Furthermore, many of these infections are caused by multidrug-resistant organisms (MDRO), which significantly hampers treatment options, extends the duration of stay, and increases cost and mortality rates 4.

Patients in the ICU are at high risk of infection due to multiple invasive procedures, malnutrition, or immunosuppression 5. Life-saving activities require the involvement of many healthcare professionals, often teams working in different departments within a hospital, which can increase the risk of infection transmission 3. Preventive measures routinely used in daily infection control may not be effective in critical situations, such as cardiopulmonary resuscitation. The rapid increase in infections with MDRO during the COVID-19 pandemic caused anxiety and a dilemma, as the rules of the sanitary regime in ICU rooms were strictly adhered to in the prevailing epidemiological situation. Moreover, additional sanitary and epidemiological procedures were developed and adapted to the department's structure. Fear of COVID-19 infection also increased the vigilance and diligence of the staff.

The combat to reduce the number of infections and pathogen transmission became a priority for ICU staff 6. Therefore, identifying MDRO reservoirs in a hospital environment is an important target for infection prevention.

This study aimed to assess whether eliminating environmental reservoirs and implementing improved procedures for patient care and decontamination and washing equipment in the ICU will reduce the incidence of infections caused by MDR strains.

## Material and Methods

### Design and settings

The study had a single-center, prospective observational design with retrospective data analysis of infections occurring in the 4th Military Clinical Hospital in Wrocław, Poland, with particular emphasis on the ICU during the COVID-19 pandemic from March 2020 to April 2022. Data were collected based on microbiological culture data and medical documentation. Therefore, ethical approval was not required because no human or animal material was involved. Cultures were routinely collected from patients admitted to the ICU. They were a rectal swab to assess the carrier of MDR bacilli, a nasal swab to assess MRSA carrier status, a urine sample for culture, in the case of intubated patients, bronchial secretions, and blood cultures if indicated. Environmental inoculations were performed during the COVID-19 pandemic on average every 4-6 weeks; unless the incidence of infections caused by MDR strains was observed, research was intensified.

### Sample collection

The samples were collected in the newly opened intensive care unit (10 stations) where COVID-19 patients were hospitalized. Samples were collected using Amies media transport swabs (Copan). Rectal swabs for carbapenemase-producing bacilli were inoculated on chromID CARBA SMART Agar (bioMerieux), chromID ESBL Agar (bioMerieux), chromID VRE Agar (bioMerieux), chromID MRSA SMART (bioMerieux) and incubated aerobically at 35-37°C for 24h. Other clinical samples (urine, BAL, nasal/throat swab, blood culture) were inoculated on appropriate culture media and incubated aerobically at 35-37°C for 24h. Swabs from the hospital environment, after 24h pre-incubation at 35-37°C, were inoculated on the following culture media: Columbia Agar (bioMerieux), Mac Conkey Agar (bioMerieux), Sabouraud Gentamicin Chloramphenicol 2 Agar (bioMerieux), chromID CARBA SMART Agar (bioMerieux), chromID ESBL Agar (bioMerieux), chromID VRE Agar, chromID MRSA SMART and incubated for another 24h under aerobic conditions at 35-37°C. Bacterial colonies were identified using VITEK2 cards, and the resistance mechanisms were determined using the disk diffusion method. An OKNVI RESIST-5 (CORIS BioConcept) immunochromatographic tests were performed from the strains insensitive to carbapenems, enabling the determination of the type of carbapenemase (KPC, NDM VIM, OXA-48, OXA-163).

### Assessment of the relationship between the strains

The clonal relationship of Klebsiella pneumoniae strains isolated from the hospital environment and from randomly selected ICU patients was determined on the basis of genomic DNA evaluation by ERIC-PCR (Enterobacterial repetitive intergenic consensus polymerase chain reaction), consisting of the use of primers complementary to highly conserved genomic DNA sequences in the amplification reaction. The Genomic Mini kit was used to isolate genomic DNA (A&A Biotechnology®, Gdańsk, Poland) **(Table 1).**

The reaction was carried out in a volume of 25μl using primers ERIC-1 and ERIC-2 (Genomed, Warsaw, Poland), purified water free from nuclease (Aqua pro inj. Polpharma®, Starogard Gdański, Poland) and a mixture of StartWarm HS-PCR Mix (A&A Biotechnology®, Gdańsk, Poland), which consisted of Taq DNA polymerase, MgCl2, deoxyribonucleotides (dNTPs), red dye and loading buffer **(Table 2)**.

The primer sequences and thermal profile of the reaction are presented in **Tables 3 and 4**. Two reference strains were used as positive controls: K. pneumoniae ATCC 4352 (W1) and K. pneumoniae ATCC 700603 (W2). The purity control of the process was purified water free of nuclease (K-).

After the completion of the polymerization reaction, the samples were electrophoresis in 2% agarose gel (Agarose LE Standard, Blirt®, Gdańsk, Poland), which enabled the visualization of highly conservative genomic DNA sequences and the analysis of the degree of kinship of the tested strains. Midori Green Direct (Nippon Genetics Japan, Tokyo, Japan®) was used as a dye, which was added directly to the samples before applying them to the gel. As a size marker, 6 μl DNA Marker 100bp (ABO Ltd., Gdańsk, Poland) with a range of 100 to 3000 base pairs was used. Electrophoresis was carried out for 2 hours at 24°C, in a single concentrated TAE buffer (Blirt®, Gdańsk, Poland), at 40V, using an electrophoresis apparatus (Cleaver Scientific, Rugby, Warwickshire, United Kingdom). The gels were taken using the OmniDOC system (Cleaver Scientific Ltd, Rugby CV22 7DH, UK).

A dice-type comparative method was used to analyze the clonal relationship of the studied strains, and similarity clusters were estimated using the UPGAMA method. For easier illustration of the results of the study, strains isolated from the environment were assigned numbers from 1 to 3, and strains from clinical materials of patients in the ICU were assigned numbers from 4 to 7. Details of the samples are presented in **Table 1**.

### Standard of nursing care in the ICU (before the Covid-19 pandemic)

Standard care of ICU patients requires a well-educated staff that knows the standards of care, performs specialized procedures, and conducts medical interventions properly. Ventilator-associated pneumonia (VAP) was the most common cause of infections in intensive care units. Extensive prophylaxis included the use of suction systems from the subglottic space, closed suction sets and pressure in the endotracheal / tracheostomy tube cuff between 20-30 cm H_2_O, raising the headrest to 45 degrees, and physical therapy. In addition, optimizing sedation at the RASS 0 level and daily sedation pauses to attempt spontaneous breathing were standard.

Before the pandemic, oral care was the preventive package against adverse events related to mechanical ventilation. Disposable packages were used - rinses with Chlorhexidine (CHG) along with brushing the teeth three times a day. If necessary, dental consultations for sanitation in the oral cavity were conducted. Inserting the low-lumen gastric feeding tube through the nose was always preceded by cleansing the nasal vestibule with a gel with disinfecting properties. To prevent urinary tract infections, the patient was catheterized with a silicone catheter connected to a closed system containing an anti-return valve. The urinary catheter was replaced every 14 days, hanging below the patient's level. Urine was collected for laboratory tests by puncturing the distal part of the catheter. The system was never disconnected.

The puncture sites of CVC were covered with transparent dressings to observe the catheter site, replaced every seven days if there was no need to replace it earlier. Each of these activities was described in the patient's file, including insertion date and catheter removal. Education on hand disinfection was also provided to patients. The full body wash was performed during night shifts in the patient's bed with a solution of water and chlorhexidine digluconate in cellulose bowls, which then were sent to the macerator for easy and safe disposal. Single-use urinals and bedpans were also disposed of in macerators, reducing contamination risk.

Daily Care Report was routinely used during the study period. It included details about the timing of replacement of disposable equipment: replacement of an infusion set for enteral nutrition, parenteral - every 24 hours, respiratory systems - every 14 days, or multi-block infusion ramps - every 72 hours. This approach eliminated mistakes in missing deadlines and was helpful in everyday activities.

The intensive care workstation was decontaminated after the patient was discharged from the ICU or passed away. Disposable fibrin wipes were used to disinfect surfaces. Areas with a special risk of exposure, medical areas, and devices were disinfected and cleaned with a broad spectrum of bactericidal (including MRSA, VRE), mycobactericidal, and fungicidal agents active against viruses (including HBV, HIV, HCV, Vaccinia, Rota, and Noro) and spores (including Clostridioides difficile).

**Figure 1:**
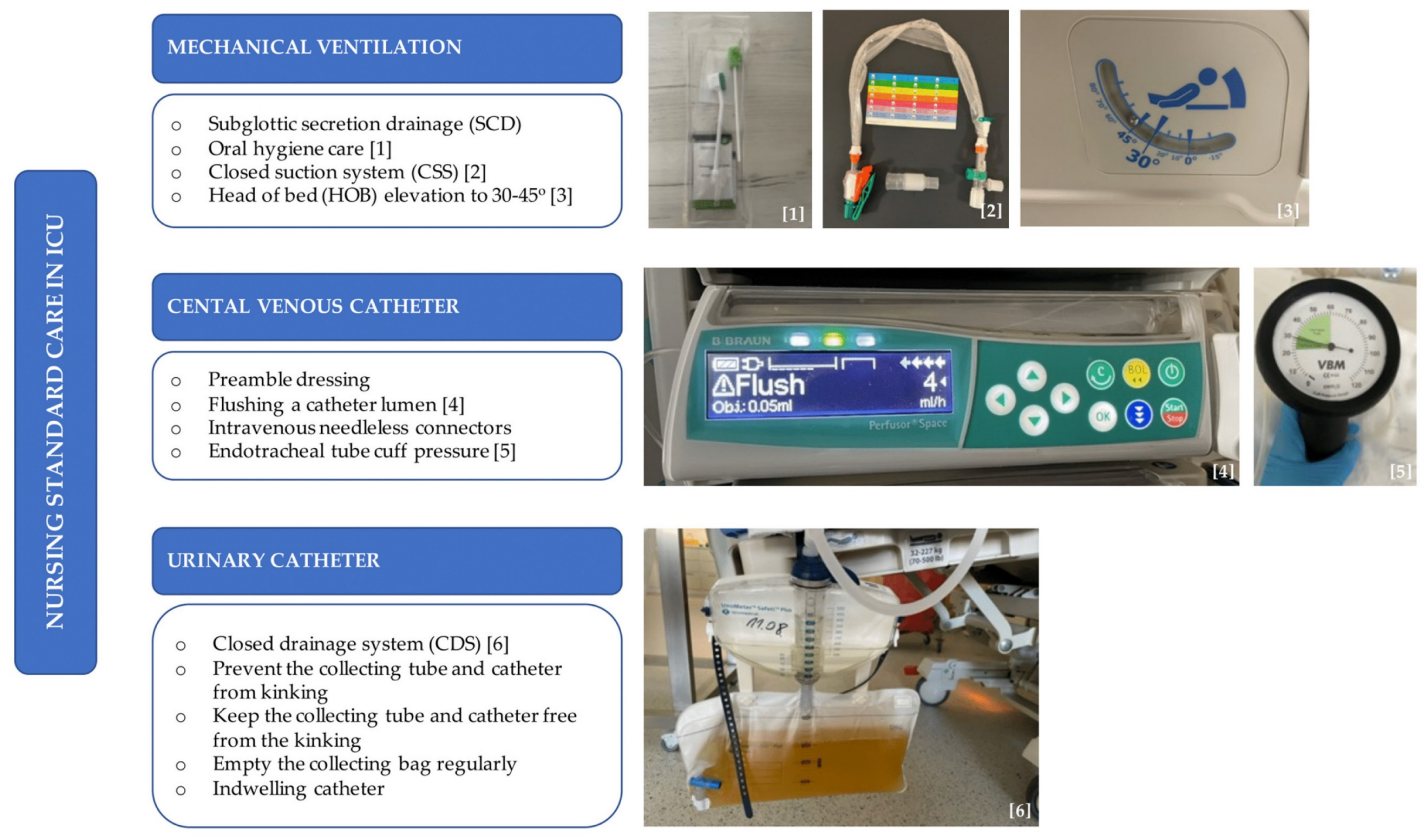
Nursing standard of care in ICU.

In the ICU, decontamination (antibacterial) mats were placed on the floor before the patient rooms. The width of the mats was adjusted to the entire rotation of the wheels while the patient's bed was moving. The arrangement of the dispensers with the hand disinfectant met World Health Organization (WHO) criteria **(Fig. 1)**.

### Bundle of Nursing Care in the COVID-19 Pandemic

The pandemic introduced new solutions and work organization rules in the hospital. In the ICU, severe cases of SARS-Cov2 infection were treated. Recommendations for the management of patients with COVID-19 were developed for the ICU staff. In addition to the basic rules, the following were introduced: disposable scrubs and caps in the area outside the wards, and special attention was paid to washing and disinfecting hands. Barrier clothing, FFP2 or P3 military masks, and double protective goggles were used. As a result, the amount of medical equipment and medicines in the ward was minimized. In addition to the patient rooms, disposable and protective equipment was stored in a specially designated area. The nursing staff stayed in the clean zone, securing the equipment and medicine needs of the staff staying in the patient rooms. This solution prevented unnecessary movement of nurses from dirty to clean zones and reduced the number of procedures performed in PPE. The possibility of virus contamination of the respiratory tract was also resolved by securing personnel in masks. Documentation was located outside the units and did not postpone their subsequent fumigation **(Fig. 1)**.

### Corrective Action in Nursing Procedures

Despite the restrictions, an increase in the frequency of infections and colonization caused by MDRO was observed. The staff work schedule during the pandemic was changed, but certain activities were performed with less intensity. The management of some of the standard anti-infection procedures was feasible due to the prone positioning of the patients. Prone positioning requires the presence and cooperation of many staff members, mainly in the case of patients requiring extracorporeal membrane oxygenation (ECMO) or continuous renal replacement therapy (CRRT), thus increasing the likelihood of infection.

The pathogen spread was analyzed, and the findings indicated procedural errors. The nurses dressed individually in multi-person rooms, but the change of their outerwear between tending to different patients was not considered. Therefore, the first decision was to secure barrier clothing with additional protective gowns when performing procedures on subsequent patients. The introduction of additional restrictions did not reduce the number of infections. Assuming that the transmission of infections through the hands of staff was significantly limited by the restrictive use of personal protective equipment, the search for a reservoir of microorganisms in the environment began.

MDR strains were grown only from the inoculations collected from the hand-wash basins in the ICU wards. These were Klebsiella pneumoniae-producing acquired NDM class B carbapenemase, Pseudomonas aeruginosa, Acinetobacter baumannii, and Escherichia coli. The basins were thoroughly decontaminated, including the spouts and siphon tanks **(Table 5)**. Bottled water for washing and drinking was used in the wards. The full body wash of patients was made only with disposable wipes with 2% Chlorhexidine; one wash wipe was applied to a specific body area and discarded.

A surprising fact was that a smear test was positive from the sample taken inside the air conditioner from a non-contact area located on the ceiling outside the patient rooms. The MDR strain of Acinetobacter baumannii was bred there. This resulted in the closure of half of the ward and subjecting it to thorough decontamination and fumigation.

Additionally, a new type of decontamination mats was used in high-risk areas, where absorbent pads were filled with Glucoprotamine solution, which is virucidal against all enveloped viruses. Active chlorine-containing substances were widely used to clean and disinfect surfaces in the wards and sanitary facilities. The rinsing of the wash basin and sanitary facilities with 10% acetic acid boiled solution was regularly repeated. In addition, hand hygiene was kept to the highest possible standard given the possibility of carrier persistence - it was assumed that many microorganisms could survive on the hands long enough to spread through the ICU environment 1.

The microbiological cleanliness of the department was monitored, and the frequency and intensity of cleaning of the patient rooms were increased. The steady, unchanging staff responsible for maintaining cleanliness and working closely with the entire team was a great convenience. It should be noted that having experienced personnel is indispensable in providing comprehensive care for a patient hospitalized in the ICU.

All ICU health professionals showed great understanding of the procedural changes and carried out the newly introduced procedures with commitment. The use of additional intervention packages and organizational changes with occasional low staffing was an additional burden. However, ready-made instructions and education of the nurses, physicians, and physiotherapists facilitated the implementation of changes in the scope of care.

Patients confirmed to be infected or colonized with MDRO were either cohorted or isolated. After the discharge or death of patients, the rooms were routinely cleaned with disinfectants, dried, and then fumigated. Fumigation consisted of fogging the rooms and their equipment with a 12% solution of hydrogen peroxide and silver ions. The patient beds were also subjected to vaporized hydrogen peroxide (VHP) gas decontamination in a decontamination chamber. This process was only one of the components of room decontamination after isolation or once the epidemiological outbreak receded. The ICU patient rooms were thoroughly cleaned at least once a month.

## Results

During the study period, an increase in the frequency of infections and colonization caused by MDRO was observed compared to the preceding years **(Table 6)**. Due to the ongoing COVID-19 pandemic, the ICU was divided into a part where patients with SARS-CoV-2 infection were treated and an area where other patients requiring ICU treatment were hospitalized. These latter patients had been admitted after surgeries or transferred from medical departments and the Emergency Unit.

During the study period, 200 hospital beds (42% of total beds) and 10 intensive care beds (100% of total beds) were dedicated to treat COVID-19 patients. A total of 23,167 tests for microbiological diagnostics and 6,985 screening tests for CPE and MRSA bacilli were collected, and 2,685 environmental microbiological tests **(Tables 7 and 8)**.

A total of 490 environmental tests were performed in ICU **(Table 9)**. The swabs were taken from critical areas due to frequent contact with patients or potential infection risk. Surfaces at the highest risk of pathogen transmission ("high-touch surfaces"), contribute to direct and indirect microorganism transmission. Samples were collected from places such as: hand-washing sinks, intravenous pumps, bed rails, bed touch panels, cardiac monitors, worktops, ventilator panels, ultrasound and X-ray machines, panels of critical parameters analyzers, and computer keyboards. Samples were taken both during periods of epidemic outbreaks and after their resolution. Periodic microbiological cleanliness checks were also conducted in the ICU. In addition, the hands of the staff were regularly inspected.

The presence of Klebsiella pneumoniae NDM strains in washbasins in 3 patient rooms was identified. In addition, the presence of Acinetobacter, Pseudomonas, Staphylococcus aureus, and other less important pathogens was demonstrated **(Table 5)**.

Data were collected in the period from March 2020 to March 2022 and were distinguished into 2 groups: before and after the introduction of the corrective action in nursing care and new methods of decontamination of the environment. Before introducing new solutions (03.2020-03.2021), the incidence was 21/1000 pd and 14.5/1000 pd for infections and colonization of NDM, respectively. After implementing additional solutions aimed at reducing the transmission of MDRO in the hospital environment, the frequency of NDM strains was significantly reduced. In the second of the analyzed periods (04.2021-04.2022), the incidence was 6.9/1000 pd and 7.5/1000 pd for infections and colonization, respectively, with the same number of hospitalizations in both analyzed periods **(Table 10)**.

After the introduction of new cleaning rules, the complete elimination of Acinetobacter sp. and Klebsiella pneumoniae NDM from the environment was demonstrated, while an increase in the presence of Pseudomonas aeruginosa was observed—assessment of the clonal relationship of environmental strains with strains detected in patients hospitalized in the ICU.

**Figure 2:**
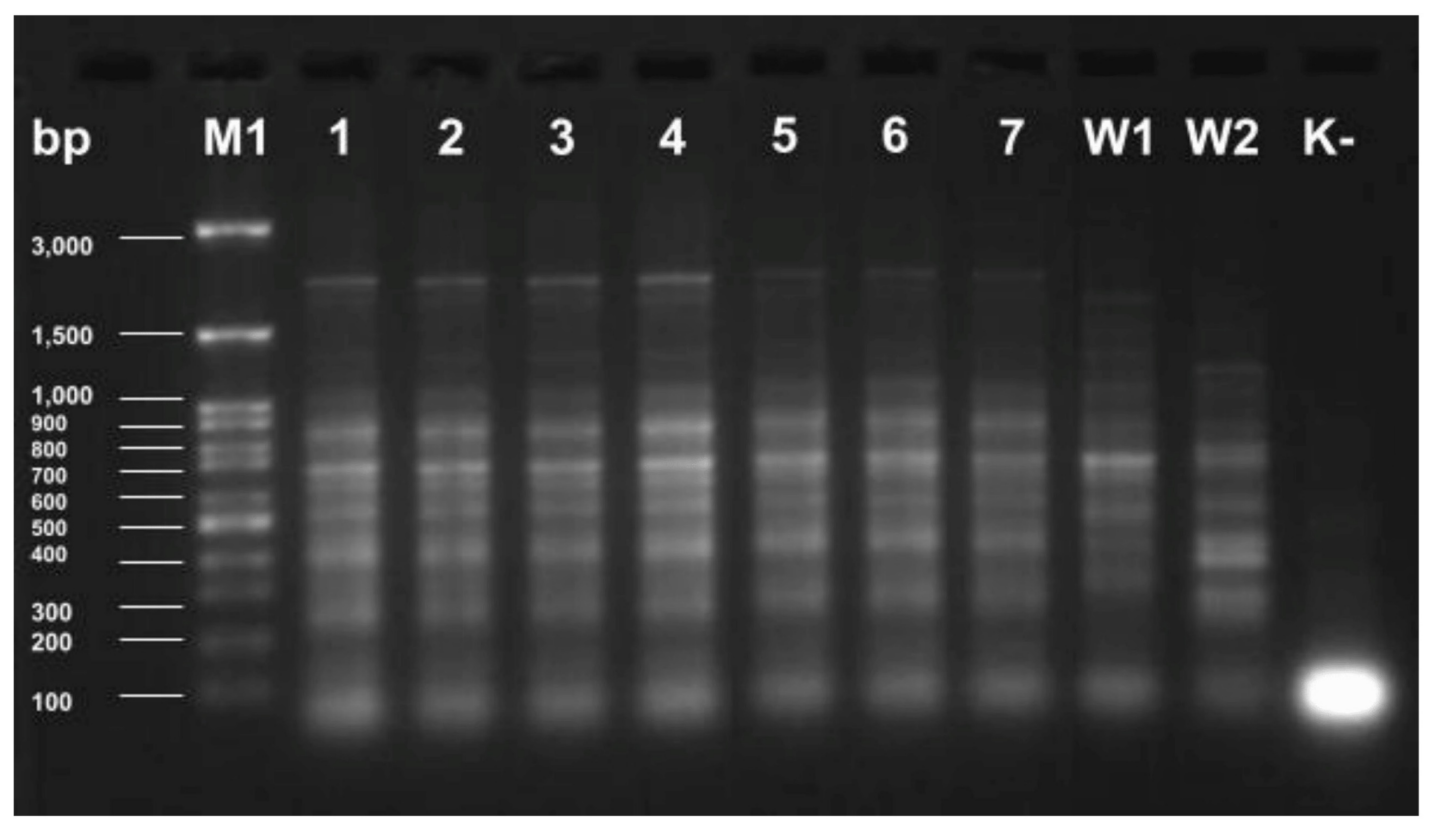
Electrophoretic separation of ERIC-PCR products of *K. pneumoniae* strains isolated from the hospital environment 1-3 and from ICU patients 4-7. W1 - *K. pneumoniae* ATCC 4352; W2 - *K. pneumoniae* ATCC 700603; K- - control of the purity of the reaction; M1 - marker size 100-3000 bp.

To assess the relationship of Klebsiella pneumoniae strains by ERIC-PCR, previously isolated genomic DNA and appropriate primers were used to replicate highly conserved genomic DNA sequences. The obtained products were electrophorized in agarose gel, showing the obtained electropherogram** (Fig. 2)**.

**Figure 3:**
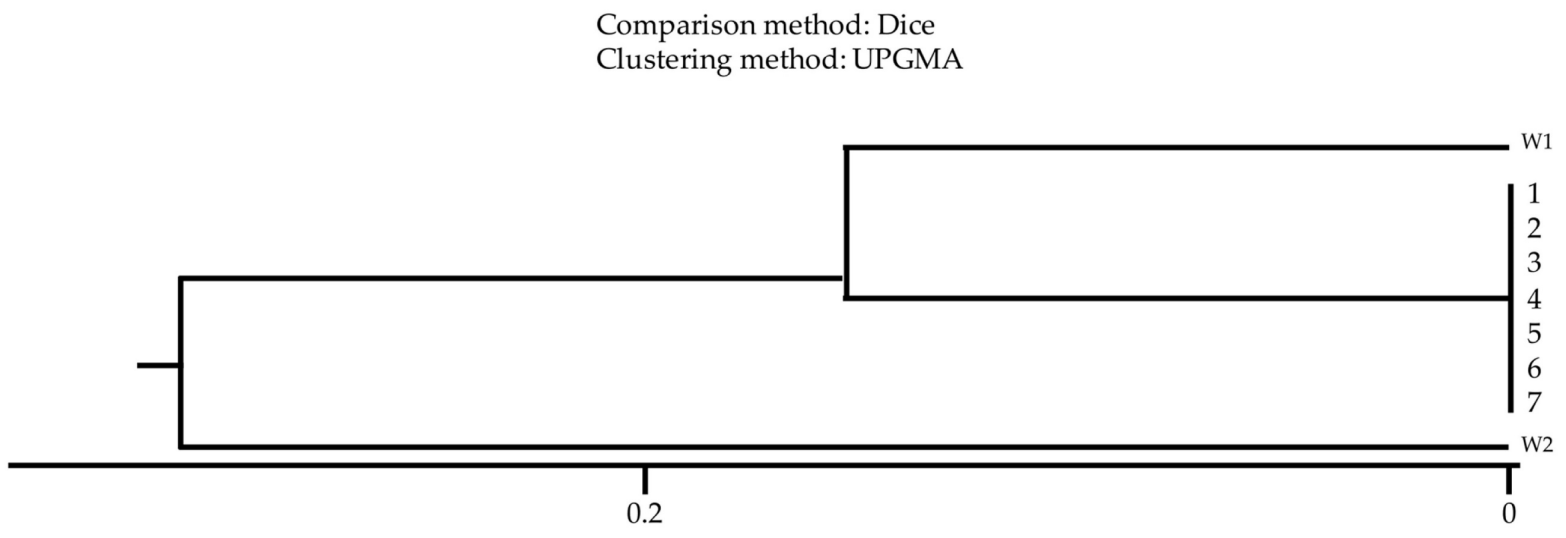
Relationship analysis of *K. pneumoniae* strains isolated from the hospital environment 1-3 and from ICU patients 4-7. W1 - *K. pneumoniae* ATCC 4352; W2 - *K. pneumoniae* ATCC 700603; Comparison method: Dice; Clustering method: UPGMA.

The analysis of clonal relationship using the comparative dice method showed that all tested strains derived from patients' clinical materials and those isolated from washbasins were characterized by a high degree of similarity, proving their origin from one clonal strain **(Fig. 3)**.

## Discussion

During COVID-19, a significant increase in infections and carriers of mainly Klebsiella pneumoniae NDM was observed. Therefore, corrective actions were taken in the field of nursing procedures, washing and cleaning of medical equipment, decontamination of beds and all workplace equipment. At the same time, the ICU environment was intensively studied to search for reservoirs. The number of infections and carriers was assessed before the implementation of additional or adjusted patient care procedures, the elimination of reservoirs, and additional cleaning of equipment. The relationship of strains located in the environment with those grown from patients was analyzed.

Before the COVID-19 pandemic, the Antimicrobial Stewardship implementation of antibiotic protection programs was successfully stepped up. With the advent of the COVID-19 pandemic, with unclear recommendations for anti-infective treatment, the use of antibiotics increased significantly despite the lack of evidence of their beneficial effects on the course of viral infection. Healthcare-associated infections (HAIs) are associated with increased mortality, morbidity, and treatment 7-10.

In the 4th Military Clinical Hospital in Wroclaw, during the pandemic, i.e., from March 2020 to April 2022, patients from the entire region were hospitalized, requiring surgical interventions and treatment in the Medical Departments or the ICU dedicated to patients with COVID-19. These patients were treated for many days in other hospitals with numerous antibiotics without current microbiological cultures. Still, the percentage of screening tests for the carrier of CPE or MRSA was negligible.

Due to the enormous number of COVID-19 patients, temporary wards were established. All patients admitted to the ICU or Medical Units underwent routine screening tests. Until the results were obtained, patients were placed on available beds, with practically no possibility of isolation from other patients with known bacterial flora. At the time of the screening test results, an attempt was made to cohort or isolate patients carrying multiple chronic conditions (MCC). Despite large restrictions in the ICU and hospital, a large spread of MDR strains was noticed. Bacterial infections that occurred during COVID-19 often worsened patients' health conditions.

Most procedures that were developed during the pandemic mainly protected the hospital personnel against COVID-19. Infected patients were served by personnel working in protective suits, which secured the staff but made it difficult to maintain the sanitary regime protecting patients - all activities with patients were performed with the same precautions, and only the gloves were changed. This created a potential risk of transmitting infectious material onto the surface of the suits.

The mechanism of colonization of the hospital environment is not fully understood 11-13. So far, several possible routes for transmitting MDR strains and settling the hospital environment have been described **(Fig. 3)** 7,14-16.

Initially, it was assumed that personnel were the main route of spreading infections due to the necessity to use personal protective equipment. However, satisfactory results were not achieved despite the application of additional care rules for patients. Additionally, frequently touching and used devices, such as an ultrasound apparatus or an X-ray machine, can be used as a route for MDRO transmission 17-21. Water and sewage systems inhabited by MDRO seem to be pivotal habits, where, at the same time, biofilms were formed. This environment is conducive to the vicious multiplication of pathogens and is extremely difficult to decontaminate 13,22-24.

Two techniques were used to check the affinity of MDRO isolate from environmental samples with patient samples ERIC-PCR and UPGAMA. The ERIC-PCR method uses primers complementary to highly conserved sequences of genomic DNA. Enterobacterial repetitive intergenic consensus sequences are 127-bp imperfect palindromes that occur in multiple copies of Enterobacterales and Vibrios genomes. They can be found between different genes; however, their number and distribution may be different between strains of the same species. ERIC-PCR technique is a quick, reliable, and cost-effective method for molecular typing of Enterobacteriaceae family members. We point out that all tested strains were the identical clones.

In the own study, places with increased humidity, such as the spouts of washbasins and the air conditioner outputs, were shown as the main reservoir. In a study published by Sukhum et al. 25, similar results were obtained: AROs were more frequently on sink drains and rarely in any other ICU rooms. In our study, it was unexpected that the air conditioner output, placed on the ceiling and out of reach of anybody, was the reservoir of MDRO. However, AROs were not shown on flat surfaces of furniture, tabletops, or even cupboard handles. Both Enterobacterales and Acinetobacter have been found in the spouts. However, the above-described pathogens were not grown from furniture, beds, or other frequently used devices, such as ultrasound apparatus.

The results obtained are useful in the context of the infection team. Currently, it is not recommended to collect environmental samples routinely. Still, if an increase in the number of infections caused by MDRO is observed, environmental swabs may be targeted as the first line of research at potential reservoirs, such as sinks, taps, or sanitary facilities. Scientific studies have shown that contaminated surfaces surrounding the patient, such as handrails and beds, may constitute a reservoir of bacteria and influence the incidence of HAI. Therefore, implementing additional cleaning and disinfection procedures may reduce the number of infections 18, 26.

After implementing a new type of decontamination, changing the routine care of patients, and quitting the use of sinks, the NDM incidence rate increased from 14.5 to 7.5 per 1000pd. In addition, a change in pathogens occurring in the environment was observed. Klebsiella pneumoniae NDM, Acinetobacter, and CoN Staphylococcus were eliminated or significantly reduced. There was an increase in the amount of Pseudomonas sp. in the environment, while these were strains without resistance mechanisms **(Table 5)**.

In the era of the growing number of multidrug-resistant bacteria, it is crucial to introduce measures to limit their spread in the hospital environment. First of all, it is essential, in the situation of an increase in the number of infections caused by MDRO, to properly collect samples from the hospital environment to assess the prevalence of MDRO in the environment surrounding the patient. Then, thanks to the ERIC-PCR method, it is possible to inform infection teams whether we are dealing with a single-disease epidemic or whether these are unrelated clones. The final element is the implementation of rules for cleaning the patient's environment to prevent the creation of bacterial reservoirs. For this purpose, new types of decontamination mats were used in high-risk areas, and active chlorine-containing substances were widely used to clean and disinfect surfaces. It is essential to create guidelines that will clearly describe hospital procedures such as proper hand hygiene, cleaning procedures, and adequately conducting an epidemiological investigation in case of the spread of infections.

## Conclusions

Identification of bacterial reservoirs and comprehensive nursing care significantly reduce the number of nosocomial infections. Without a doubt, the ICU should be a place with extensive epidemiological restrictions. Hospitalized patients often have significantly reduced immunity, numerous stresses, or disturbances in the postoperative period. Therefore, extensive efforts have been made to discover and eliminate reservoirs. Such activities should be applied to all departments as a routine procedure, and the collection of cultures as part of environmental studies from potential reservoirs should be implemented. The investigation and action implemented in our hospital have eliminated the hazard of spreading the MDR strains among ICU patients.

## Figures and Tables

**Table 1:** Strains of Klebsiella pneumoniae subjected to clonal relationship analysis.

No.	Date of insulation	Insulation material	Age of patient
1	01/04/2021	Washbasin 1	-
2	01/04/2021	Washbasin 2	-
3	01/04/2021	Washbasin 3	-
4	08/03/2021	Blood	21
5	17/03/2021	Pus	56
6	25/03/2021	BAL	61
7	29/03/2021	Swab	64

**Table 2:** Components needed for the analysis of strains by ERIC-PCR.

Component	Volume (μl)
StartWarm HS-PCR mix	12.5
Water miliQ	8.5
Primer 1	1.0
Primer 2	1.0
DNA Genom (matrix)	2.0

**Table 3:** Primers used for the analysis of strains by ERIC-PCR.

Primer	Amino acid sequence (5'-3')
ERIC-1	ATG TAA GCT CCT GGG GAT TCAT
ERIC-2	AAG TAA GTG ACT GGG GTG AGCG

**Table 4:** Thermal profile of the ERIC-PCR method.

Stage		Temperature [°C]		Time
Preliminary denaturation		95		7 min.
Denaturation	Number of cycles	90	30 CYCLES	30 sec.
Annealing		52		1 min.
Synthesis		65		8 min.
Final polymerization		65		16 min.

**Table 5:** Bacterial strains cultured in environmental tests in the ICU.

Bacterial strain	03.2020-03.2021	04.2021-04.2022
Acinetobacter spp.	5	0
Bacillus spp.	20	2
CoN Staphylococcus	16	1
Escherichia coli	1	0
Klebsiella pneumoniae	1	0
Klebsiella pneumoniae NDM	3	0
Kluyvera intermedia	1	0
Pseudomonas aeruginosa	4	6
Pseudomonas putida	1	0
Sarcina sp.	7	0
Staphylococcus aureus MSSA	1	0

**Table 6:** Number of NDM cases in the ICU.

Period:	03.2018-03.2019	03.2019-03-2020	03.2020-03.2021	04.2021-04.2022
Carriage	4	1	50	26
Infection	4	3	73	24

**Table 7:** Screening microbiology tests in a period 03.2020-04.2022 in patients treated in the 4th Military Clinical Hospital of Wroclaw.

Screening microbiology tests	N
Rectal swab	4,862
Throat swab	577
Nasal swab	1,546
Total	6,985

**Table 8:** Environmental microbiology tests in between 03.2020-04.2022 in the 4th Military Clinical Hospital of Wroclaw.

Environmental microbiology tests	N
Surface cleanliness test	2,374
Air cleanliness testing	174
Endoscope cleanliness testing	132
Total	2,680

**Table 9:** Environmental microbiology test in a period 03.2020-04.2022 in patients treated in the ICU.

Environmental microbiology test	N
Surface cleanliness test	489
Air cleanliness test	1
Total	490

**Table 10:** Incidence of NDM in the ICU.

Period:	03.2020-03.2021	04.2021-04.2022
Carriage	21/1000 pd	6.9/1000 pd
Infection	14.5/1000 pd	7.5/1000 pd
Number of hospitalizations	426	445

## Abbreviations

CAESAR: Central Asian and European Surveillance of Antimicrobial Resistance
CHG: Chlorhexidine
CRRT: continuous renal replacement therapy
EARS-Net: European Antimicrobial Resistance Surveillance Network
ECMO: extracorporeal membrane oxygenation
ICU: intensive care unit
MCC: multiple chronic conditions
MDRO: multidrug-resistant organisms
VAP: ventilator-associated pneumonia
VHP: vaporized hydrogen peroxide
WHO: World Health Organization
